# How do core personality traits influence short video dependence among Chinese college students? Evidence from a serial mediation analysis under the I-PACE model

**DOI:** 10.3389/fpsyg.2026.1763608

**Published:** 2026-02-25

**Authors:** Jin Zhang, Lulu Ren, Yaxin Wu, Yafei Shi, Jinhai Liu

**Affiliations:** 1 School of Mathematics and Statistics, Henan Normal University, Xinxiang, China; 2 Faculty of Education, Henan Normal University, Xinxiang, China

**Keywords:** college students, fear of missing out, I-PACE model, perceived self-efficacy, serial mediation, short-video dependence

## Abstract

**Background:**

With the widespread adoption of short videos, college students have developed an unprecedented level of dependence, posing significant challenges to society. Existing research on short-video dependency (SVD) has primarily focused on behavioral symptoms; however, the role of core personality traits (CPT) and their underlying cognitive–emotional mechanisms in predisposing individuals to SVD remains insufficiently understood.

**Objective:**

This study aims to examine the relationship between CPT and SVD and to clarify the cognitive-emotional pathways underlying this association by extending the Interaction of Person-Affect-Cognition-Execution (I-PACE) framework to include perceived self-relatedness (PSR) as an additional mediator.

**Methods:**

Survey data were collected from 825 Chinese undergraduate students. Structural equation modeling was used to test an enhanced serial mediation model incorporating CPT, fear of missing out (FoMO), perceived self-efficacy (PSE), PSR, and SVD.

**Results:**

Core personality trait, FoMO, and PSR were correlated with SVD. In contrast, PSE showed an inconsistent mediation pattern, exhibiting a positive bivariate correlation but a negative indirect effect. FoMO, PSE, and PSR significantly mediate the relationship between CPT and SVD. The identification of this inconsistent mediation provides evidence for dual-process competition in short-video use behavior.

**Conclusion:**

The findings suggest that the risk of SVD is statistically associated with lower levels of FoMO, higher PSE, and regulating exposure to self-relevant content among students. By highlighting the roles of cognitive and emotional factors, this study advances understanding of the antecedents of SVD and offers theoretical and practical implications for future research and intervention strategies.

## 1 Introduction

With the widespread adoption of mobile internet and the increasing demand for fragmented information consumption, short-form video platforms have emerged as a major channel for accessing daily information and entertainment (Hu et al., 2024). Characterized by short duration and diverse content formats, short-form videos typically range from a few seconds to several minutes in length (Yuan and Wang, 2024). Their fast-paced and visually stimulating nature aligns well with fragmented viewing contexts and facilitates rapid emotional engagement among users (Kaye et al., 2021). Recent statistics indicate that TikTok has approximately one billion active users worldwide. The platform reports an average daily usage time of 95 min per user, ranking among the highest across major social media platforms (Ceci, 2025). In China, the scale of short-form video use is even more pronounced. According to the 56th Statistical Report on Internet Development released by the China Internet Network Information Center (CNNIC), by June 2025 China had 1.123 billion internet users, of whom 1.068 billion were short-form video users, accounting for 95.1% of the total online population (China Internet Network Information Center, 2025).

Meanwhile, short-form video platforms continue to expand their commercial and functional ecosystems. An increasing number of digital media services now integrate short-video features to enhance user engagement (Wang T. Y. et al., 2025; Wang X. et al., 2025). Existing research suggests that short-form videos may offer several functional benefits. For example, short-form videos have been shown to alleviate academic stress among students (Ramsden and Talbot, 2024), support informal and micro-learning opportunities (Handorf et al., 2024), and assist parents in identifying educational applications and resources (Pearson et al., 2023). However, excessive engagement with short-form video content has raised increasing concerns. Many students report difficulties in regulating their viewing behavior. Such difficulties may lead to attentional impairments, deficits in behavioral inhibition, and the development of short-video dependence (SVD) (Jiang A. et al., 2025; Jiang Y. et al., 2025). SVD has been associated with a range of adverse outcomes, including reduced learning motivation (Ye et al., 2022), lower academic engagement, increased attentional distraction (Chen et al., 2023; Hong et al., 2014), and distorted time perception (Jiang A. et al., 2025; Jiang Y. et al., 2025). These negative consequences underscore the need for systematic investigation of the underlying mechanisms of SVD, which is essential for informing evidence-based interventions and promoting healthier patterns of digital media use (Roberts and David, 2025).

Previous studies have sought to explain SVD from multiple theoretical perspectives. Some studies emphasize platform-level factors, such as recommendation algorithms, which can shape users’ viewing patterns and weaken self-regulatory control over media consumption (Wang et al., 2024). Other studies highlight contextual influences, suggesting that socially anxious environments may exacerbate students’ vulnerability to SVD (Sert and Baskale, 2023). From a motivational perspective, studies grounded in Uses and Gratifications Theory indicate that students frequently engage with short-video platforms to satisfy specific psychological needs, including entertainment, escapism, and social connection (Zhang M. et al., 2023; Zhang N. et al., 2023). In addition, reinforcement-based explanations suggest that instant gratification and algorithm-driven positive feedback loops reinforce habitual and repetitive viewing behaviors (Liu et al., 2024). Despite these contributions, existing research has largely focused on isolated external or motivational factors, while paying limited attention to the dynamic psychological processes that link individual predispositions with emotional responses and executive control mechanisms. As a result, the core process through which personality-related vulnerabilities are translated into emotion-driven repetitive usage patterns remains insufficiently understood. Addressing this gap is essential for developing a more integrative theoretical framework to explain SVD and for identifying key leverage points for prevention and intervention.

This study adopts the I-PACE model as its theoretical framework and focuses on college students as the target population. This study aims to examine how specific psychological traits (CPT), encompassing needs, motives, and values, manifesting in the context of short-video use are associated with SVD through key psychological mechanisms, such as fear of missing out (FoMO) and perceived self-efficacy (PSE) (Liu J. et al., 2025; Liu Y. et al., 2025). The core analytical pathway of this study is conceptualized as: CPT → psychological mediating processes → SVD. By applying the I-PACE model to integrate CPT with cognitive and affective factors, this study seeks to provide deeper insights into the mechanisms underlying SVD among college students. The specific research questions are as follows:

*RQ1*: How do CPT influence SVD among Chinese college students through the mediating roles of FoMO and PSE?

*RQ2*: To what extent does perceived self-relevance (PSR) serve as an additional mediating pathway between CPT and SVD beyond the established FoMO-PSE mechanism?

## 2 Literature review and hypotheses

### 2.1 The interaction of the person-affect-cognition-execution model

The Interaction of the I-PACE model, proposed by Brand et al. (2016), provides a theoretical framework for understanding specific internet use disorders by emphasizing CPT, emotional and cognitive responses, and individual executive function factors. The model explains how excessive internet dependence and use disorders develop through the interaction of multiple psychological and behavioral factors. Furthermore, Brand et al. (2019) refined the model by distinguishing core traits into general predisposing variables (e.g., genetic factors) and specific behavioral predisposing variables (e.g., specific needs). The model posits that individuals’ personality traits shape their cognitive and emotional responses to internal and external stimuli (e.g., internet-related cues), which in turn influence executive functioning.

The I-PACE model has been widely adopted across a broad range of empirical studies. Jhone et al. (2021) reported that adolescents with left-behind experiences are at increased risk of online game addiction due to maladaptive cognition patterns. Ye et al. (2024) found that inertia-prone college students are more likely to develop dependency on ChatGPT use when experiencing positive affect. Kim et al. (2024) argued that adolescents’ family status exerts significant indirect effects on smartphone addiction through emotional responses such as anxiety and FoMO. Academic discussions on smartphone use disorder, online game addiction disorder, and general internet use disorder has reached considerable depth; however, research on excessive short-video use remains limited (Hasan et al., 2018). Using the I-PACE model, Nong et al. (2023) found that students are more prone to developing addictive short-video use when experiencing high levels of immersion during viewing. However, these studies have not sufficiently examined the dynamic emotional and cognitive responses that emerge during the development of disordered behaviors. Based on the I-PACE model, this study explores the influence of CPT on SVD, focusing on psychological factors such as FoMO, and PSE and PSR.

Within the specific context of short-video, the present study examines the influence of CPT on SVD, with a focus on psychological factors including FoMO, PSE, and PSR. Within the specific context of short-video use, the present study operationalizes the P-component of the I-PACE model through specific behavioral predisposing variables. These variables include specific needs, motives, and values. On the one hand, these variables are grounded in general predisposing factors; for example, individuals with high social anxiety may develop a specific need for low-cost social connections through online environments (Lacombe et al., 2024). On the other hand, they contextualize general vulnerability by anchoring it to the short-video domain, thereby transforming broad predispositional tendencies into targeted psychological orientations that directly drive short-video use (Hong et al., 2025). Among the college student, the formation of specific needs, motives, and values is strongly shaped by key developmental tasks within the short-video context, including academic stress management, social exploration, and identity construction. For instance, college students’ widespread need for fragmented time management often combines with motives to avoid boredom and efficiently use spare time, giving rise to the value belief that short-videos represent an optimal medium for fragmented consumption (Xie et al., 2025). Similarly, the strong desire for self-expression and social recognition during late adolescence is transformed — through the immediate feedback mechanisms of short-video platforms — into a specific motive to obtain social validation via short-video use. This motive is further reinforced by the pervasive need for belongingness among college students (Rasmussen et al., 2020). Taken together, the transition from general vulnerability to domain-specific behavioral engagement among college students reflects the core function of needs, motives, and values as specific behavioral predisposing variables. This process closely aligns with the central logic of the I-PACE model, which posits that core personal predispositions function as foundational drivers of addictive behavioral development.

### 2.2 Core personality traits and short-video dependence

The I-PACE model posits that core personal factors constitute the starting point for the development of specific behavioral dependencies. It further emphasizes that specific behavioral predisposing variables represent key psychological orientations driving the formation of stable behavioral patterns in particular usage contexts. These predispositions are primarily manifested through individuals’ needs, motives, and value cognitions within specific media-use environments (Brand et al., 2019). In the present study, CPT are conceptualized as personality-related dispositional orientations rather than classical trait taxonomies (e.g., the Big Five), reflecting relatively stable motivational and value-based tendencies embedded in media-use contexts. Within the specific behavioral domain of short-video use, these behavioral predisposing variables constitute the proximal psychological foundation of dependency formation. Existing research has consistently demonstrated that users’ context-specific needs, motivational drivers, and value perceptions are closely associated with both the emergence and maintenance of dependent media-use behaviors. For example, satisfaction of entertainment and social needs has been shown to exert a positive effect on SVD (Zhu et al., 2024). Users engage with short-videos for specific motivations, such as escapism and stress relief, and these motivations show significant positive associations with short-video use (Chen et al., 2024). The perceived value of short-video functions (e.g., social interaction) can enhance users’ motivation to accumulate social capital, thereby increasing both the frequency and duration of short-video use (Xie et al., 2025).

Therefore, the present study conceptualizes specific behavioral predisposing variables as an integrated system of psychological orientations. Rather than representing a simple projection of general personality traits, this construct reflects a relatively stable state of behavioral readiness that is actively perceived and internalized within the specific media ecology of short-video platforms. This system serves as a bridge between macro-level personal predispositions and micro-level behavioral engagement, directly shaping the manner, intensity, and emotional attachment of users’ interactions with short-video platforms. Through the synergistic interplay of needs, motives, and values, these predisposing variables translate latent vulnerability into concrete behavioral patterns associated with dependence risk. Compared with traditional measurements of core personality traits, the specific behavioral predisposing variable framework demonstrates greater contextual sensitivity and explanatory precision. This framework shifts the analytical focus toward individuals’ psychological orientations within the short-video usage context, thereby more accurately capturing proximal and modifiable psychological mechanisms that trigger dependent behavior. Based on this theoretical foundation, the following hypotheses are proposed:

*H1*. CPT have a positive impact on SVD.

### 2.3 The mediating role of fear of missing out on short-videos

Fear of missing out (FoMO) refers to a pervasive apprehension that others may be having rewarding experiences from which one is absent, accompanied by a strong desire to remain continuously connected to online content and social information (Zhang M. et al., 2023; Zhang N. et al., 2023). In short-video contexts, FoMO typically manifests as compulsive monitoring, frequent content refreshing, and repeated platform-checking behaviors, driven by concerns about missing trending topics, viral content, or socially salient information streams (Elhai et al., 2025). A growing body of empirical evidence has demonstrated a robust positive association between FoMO and SVD, as well as broader forms of social media addiction (Chang et al., 2025). Individuals with elevated FoMO tend to engage in repetitive checking behaviors to reduce uncertainty and social anxiety. Over time, these behaviors may reinforce habitual usage patterns and weaken self-regulatory control (Huang et al., 2025). From a dispositional perspective, FoMO is often linked to heightened needs for social reassurance, external validation, and immediate gratification. These tendencies are commonly rooted in insecurity, identity-related concerns, and unmet belongingness needs (Blachnio et al., 2025). Short-video platforms further amplify FoMO through algorithm-driven recommendation systems that emphasize immediacy and popularity, thereby intensifying users’ perceived urgency to remain continuously connected (Elhai et al., 2025). In the present study, FoMO is conceptualized as a usage-related affective state activated by context-specific dispositional orientations, rather than a stable personality trait. Individuals with stronger short-video-related needs, motives, and value orientations are more likely to exhibit heightened sensitivity to content-scarcity cues and social comparison signals. This sensitivity, in turn, increases emotional arousal and reinforces compulsive engagement patterns. Although FoMO has been widely linked to problematic media use, its mediating role in translating contextualized predispositions into SVD has received limited systematic attention. Accordingly, grounded in the I-PACE model, the present study proposes that FoMO functions as a key affective mediator linking CPT to SVD.

*H2*. FoMO is a mediator in the association between CPT and SVD.

### 2.4 The mediating role of perceived self-efficacy

Perceived self-efficacy (PSE) refers to individuals’ beliefs in their capacity to regulate their behavior and cope with task-related demands (Bandura, 1986). In the present study, PSE is conceptualized as a usage-related cognitive evaluation shaped by repeated interactions with short-video platforms, rather than a stable personality trait. Within digital media contexts, users continuously update their self-regulatory judgments based on feedback cues, perceived control experiences, and platform affordances. As a result, PSE functions as a dynamic and context-sensitive psychological state (Hamann et al., 2024).

Empirical evidence has consistently shown that PSE is closely associated with problematic media use and self-regulatory outcomes. Individuals with lower PSE are more likely to rely on short-video platforms and other forms of digital entertainment to cope with stress and negative emotions, thereby increasing vulnerability to dependent usage patterns (Liao, 2024). In contrast, higher PSE is associated with greater behavioral persistence, more adaptive coping strategies, and a stronger willingness to engage in goal-directed activities, which may protect against excessive media consumption (He and Wong, 2022). Conversely, repeated engagement in addictive behaviors may further undermine perceived self-regulatory capacity, thereby creating a negative feedback cycle that reinforces dependence (Young et al., 2014).

From an I-PACE perspective, context-specific predispositional orientations (e.g., short-video-related needs, motives, and value beliefs) shape how users evaluate their ability to control platform engagement. Strong motivational involvement and value alignment with short-video use may alter users perceived behavioral control, thereby shaping subsequent usage decisions. Accordingly, the present study proposes that PSE functions as a key cognitive mediator linking core personality predispositions to SVD.

*H3*. PSE mediates the relationship between CPT and SVD.

### 2.5 The mediating role of perceived self-relevance

Perceived self-relevance (PSR) refers to individuals’ subjective evaluation of the personal significance, value, and identity-related importance of information or activities (Jin et al., 2019). In digital media contexts, PSR is conceptualized as a usage-related cognitive–affective state that emerges through repeated interactions with personalized content environments, rather than a stable personality disposition. When users encounter content aligned with their interests, values, or self-concept, they are more likely to allocate attentional resources, experience emotional resonance, and perceive greater subjective meaning in platform engagement. Prior research indicates that self-relevant processing plays a central role in shaping motivation, engagement intensity, and decision commitment. Individuals tend to preferentially attend to and remember content congruent with their identity and personal values. This process enhances emotional involvement and reinforces repeated exposure patterns (Lew and Flanagin, 2024). Moreover, self-relevant information is more likely to be shared and socially reinforced, thereby further strengthening users’ perceived personal connection to platform content (Scholz et al., 2020). Neurocognitive evidence further suggests that self-related processing is associated with increased engagement-related neural activation, supporting a link between perceived relevance and motivational salience (Scholz et al., 2023).

From an I-PACE perspective, context-specific dispositional orientations shape the degree to which users perceive short-video content as self-relevant. Individuals with stronger short-video-related needs, motives, and value beliefs are more likely to interpret platform content through an identity-congruent lens, thereby increasing perceived relevance and strengthening emotional attachment to the platform. This cognitive resonance mechanism may promote sustained engagement and contribute to the development of dependent usage patterns. Accordingly, the present study proposes that PSR functions as a cognitive mediator linking core personality predispositions to SVD.

*H4*. PSR mediates the relationship between CPT and SVD.

### 2.6 The serial mediating role of fear of missing out and perceived self-relevance

According to the I-PACE model, individual’s dependence on social media is largely shaped by CPT (Ding et al., 2024). FoMO, as a key factor contributing to addictive behavior, prompts individuals to continuously engage with social content to avoid social isolation (Yam et al., 2025). The fragmented nature of short-video content, together with the surrounding social environment, readily triggers feelings of missing out or disconnection, thereby intensifying FoMO toward short videos. Additionally, PSE has been identified as a key protective factor against the development of addiction (Tian et al., 2022). Low PSE weakens an individual’s ability to regulate their behavior, making it difficult for them to overcome dependence on short-video use. This addictive tendency intensifies when users feel unable to control their viewing time, which in turn further increases the frequency of short-video use (Alt, 2018).

Research has shown that FoMO and PSE exhibit a mutually reinforcing relationship, whereby higher levels of FoMO further reduce individuals’ PSE, trapping them in a passive usage cycle (Przybylski et al., 2013; Wegmann et al., 2017). Conversely, lower PSE can exacerbate individuals’ FoMO, thereby leading to a vicious cycle of addictive behavior (Blackwell et al., 2017). In summary, FoMO and PSE may operate as sequential mediators in the development of SVD. Based on this reasoning, the following hypothesis is proposed:

*H5*. FoMO and PSE act as serial mediators in the association between CPT on SVD.

**Figure 1:**
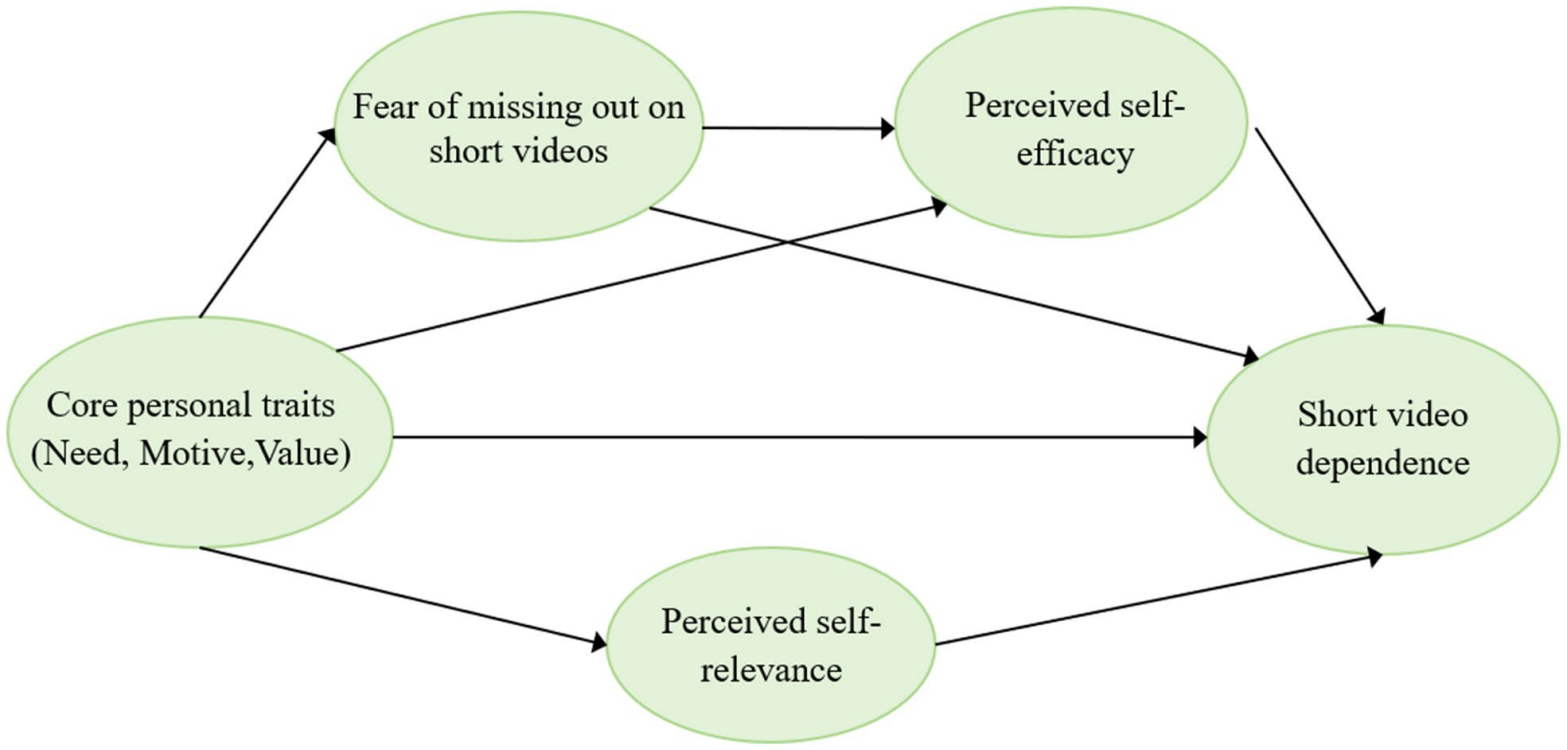
Overview of the proposed research models.

Previous research has shown that external contextual inputs shape behavioral outcomes through dynamic psychological and cognitive processes, highlighting the importance of modeling intermediate mechanisms rather than relying solely on direct-effect assumptions (Ning et al., 2026). In summary, this study constructs a sequential mediation model grounded in the I-PACE framework to examine the relationships among CPT, SVD, PSR, FoMO, and PSE (see Figure 1). Specifically, this study investigates the mechanistic pathways through which CPT influence SVD, thereby providing evidence-based support for the design of targeted behavioral interventions.

## 3 Materials and methods

### 3.1 Participants and procedure

Participants were recruited from a comprehensive university in central China using a cluster sampling strategy. Data were collected via an online questionnaire platform^1^ between July and August 2025. The survey link and informed consent form were distributed through official WeChat class groups across different academic years and majors. Participation was voluntary and anonymous. All respondents provided informed consent prior to participation and were informed of their right to withdraw at any time without penalty. The study procedures complied with the Declaration of Helsinki and were approved by the Ethics Committee of Henan Normal University.

A maximum target sample size of 900 responses was pre-specified. During data screening, responses were excluded if they met any of the following criteria: (a) completion time shorter than 60 s or longer than 600 s; (b) duplicate submissions identified based on identical response patterns and device information; or (c) uniform response patterns in which the same response option was selected for all items. After applying these quality control procedures, 825 valid questionnaires were retained for analysis, yielding an effective response rate of 91.67%.

Participants ranged in age from 18 to 25 years (*M* = 21.70, SD = 0.98). The sample included 513 females (62.2%) and 312 males (37.8%). Regarding academic year, 194 participants were freshmen (23.5%), 308 were sophomores (37.4%), 153 were juniors (18.5%), and 170 were seniors or above (20.6%). In terms of academic disciplines, 12.5% majored in science, 25.4% in technology, 18.7% in engineering, 31.3% in mathematics, and 12.1% in other fields. With respect to short-video usage frequency, 24.4% of participants reported occasional use, 29.9% reported moderate use, and 45.7% reported frequent use. Regarding daily usage duration, 19.1% reported using short-videos for less than 1 h per day, 24.4% for 1–2 h, 35.5% for 3–4 h, and 21.0% for more than 4 h (Table 1).

**Table 1:** Participants’ background information.

Demographics	Sample (*N* = 825)	
	*N*	*%*
Gender		
Male	312	37.8
Female	513	62.2
Age		
<20 years old	203	24.6
21–23 years old	447	54.2
>24 years old	175	21.2
Grade		
Freshman	194	23.5
Sophomore	308	37.4
Junior	153	18.5
Senior or above	170	20.6
Subject		
Science	103	12.5
Technology	210	25.4
Engineering	154	18.7
Mathematics	258	31.3
Others	100	12.1
Frequency		
Occasionally	201	24.4
Sometimes	247	29.9
Often	377	45.7
Usage time		
<1 h	158	19.1
1–2 h	201	24.4
3-4 h	293	35.5
>4 h	173	21.0

### 3.2 Measures

The questionnaire consisted of two sections. The first section collected participants’ demographic and usage-related information, including gender, age, academic year, major, frequency of short-video use, and average daily usage duration. The second section contained measurement items assessing the five core constructs of the study: CPT, FoMO, PSE, PSR, and SVD. Prior to the main survey, a pilot study was conducted to evaluate the psychometric properties of the adapted and newly developed scales. The results indicated satisfactory cultural suitability, reliability, and construct validity, meeting the methodological requirements of the present study. The complete measurement instruments are provided in the Supplementary material.

#### 3.2.1 Core personality traits

The CPT measurement were measured using a 12-item scale adapted from Deci and Ryan (2000) and Przybylski et al. (2013). The instrument was designed to assess dispositional orientations underlying Chinese college students’ short-video use behavior and comprised three subdimensions: Need (NE; 4 items), Motive (MO; 4 items), and Value (VA; 4 items). Representative items included “Using short-video platforms gives me a sense of accomplishment when I discover useful content” (Need), “I watch short-videos to keep up with trending topics and current events” (Motive), and “Watching short-videos helps me generate new ideas and think more independently” (Value). All items were rated on a five-point Likert scale ranging from 1 (strongly disagree) to 5 (strongly agree), with higher scores indicating stronger needs, greater motivational tendencies, and higher value orientations toward short-video use. In the present study, the internal consistency reliability of the three subscales was satisfactory, with Cronbach’s alpha coefficients of 0.939 for Need, 0.934 for Motive, and 0.952 for Value.

#### 3.2.2 Fear of missing out

The FoMO was measured using a 5-item scale adapted from Przybylski et al. (2013). The instrument was used to assess the extent to which Chinese college students experienced anxiety or discomfort related to missing updates or content on short video platforms. A representative item was “I feel anxious when I have not checked short video platforms for a while.” All items were rated on a five-point Likert scale ranging from 1 (strongly disagree) to 5 (strongly agree), with higher scores indicating higher levels of FoMO. In the present study, the scale demonstrated excellent internal consistency reliability, with a Cronbach’s alpha coefficient of 0.941.

#### 3.2.3 Perceived self-efficacy

The PSE was measured using a 4-item scale adapted from García-Salirrosas et al. (2025) and Pumptow and Brahm (2021). The scale was designed to assess Chinese college students’ perceived confidence in their ability to regulate and control short video use behaviors. A representative item was “I believe I can control the amount of time I spend watching short videos each day.” All items were rated on a five-point Likert scale ranging from 1 (strongly disagree) to 5 (strongly agree), with higher scores indicating higher levels of PSE. In the present study, the scale demonstrated strong internal consistency reliability, with a Cronbach’s alpha coefficient of 0.924.

#### 3.2.4 Perceived self-relevance

The PSR was measured using a 4-item scale adapted from Klimmt et al. (2018) and Lee and Watkins (2016). The scale was designed to assess the extent to which short video content is perceived as personally relevant by Chinese college students. A representative item was “The short videos I watch typically reflect my personal interests.” All items were rated on a five-point Likert scale ranging from 1 (strongly disagree) to 5 (strongly agree), with higher scores indicating higher levels of perceived self-relevance. In the present study, the scale demonstrated excellent internal consistency reliability, with a Cronbach’s alpha coefficient of 0.959.

#### 3.2.5 Short video dependence

The SVD was measured using a 4-item scale adapted from Ye J. et al. (2025) and Ye J.-H. et al. (2025). The scale was designed to assess the extent of compulsive and uncontrollable short video use behaviors among Chinese college students. A representative item was “I find it difficult to stop watching short videos, even when I need to do something else.” All items were rated on a five-point Likert scale ranging from 1 (strongly disagree) to 5 (strongly agree), with higher scores indicating higher levels of SVD. In the present study, the scale demonstrated strong internal consistency reliability, with a Cronbach’s alpha coefficient of 0.928.

### 3.3 Analytical approach

Data analyses were conducted using SPSS 26.0 and SmartPLS 4. Prior to hypothesis testing, data quality was assessed through descriptive statistics (means and standard deviations), normality diagnostics (skewness and kurtosis), and bivariate correlation analyses. Potential common method variance (CMV) was initially examined using Harman’s single-factor test as a preliminary diagnostic procedure.

Measurement model evaluation was performed in SmartPLS 4 using confirmatory factor analysis. Internal consistency reliability was assessed using Cronbach’s alpha and composite reliability (CR). Convergent validity was evaluated based on standardized factor loadings and average variance extracted (AVE). Discriminant validity was examined using both the Fornell–Larcker criterion and the heterotrait–monotrait ratio (HTMT), with 95% bias-corrected confidence intervals generated through 5,000 bootstrap resamples (Fornell and Larcker, 1981).

The PLS-SEM was selected for several methodological considerations. First, the proposed research model involves multiple latent constructs and complex serial mediation pathway that may exhibit inconsistent mediation patterns. Second, our analytical approach followed contemporary guidelines for testing and interpreting inconsistent mediation effects (Hayes, 2018; Zhao et al., 2010), including the examination of competing indirect effects and their theoretical implications. Third, this study adopts a prediction-oriented perspective aimed at theory extension within the emerging research context of SVD, for which PLS-SEM has been widely recommended. In addition, PLS-SEM is relatively robust to minor deviations from multivariate normality and demonstrates strong performance in estimating complex structural models (Liu J. et al., 2025; Liu Y. et al., 2025). To further assess potential CMV-related bias, variance inflation factor (VIF) values were examined in the structural model to detect collinearity effects. All VIF values were below conservative threshold levels, indicating that CMV was unlikely to substantially influence the estimated relationships (Chen et al., 2025a; Chen et al., 2025b).

Structural model estimation was subsequently conducted to test the hypothesized direct and indirect relationships. A nonparametric bootstrapping procedure with 5,000 resamples was applied to estimate standard errors and bias-corrected confidence intervals for path coefficients and mediation effects. Sequential mediation effects involving FoMO and PSE were evaluated based on these bootstrapped confidence intervals. In addition, to verify the stability of the model conclusions, this study subsequently conducted a robustness test of the structural equation model by incorporating covariates such as gender and age via AMOS, so as to control for the potential confounding effects of demographic and behavioral variables.

## 4 Results

### 4.1 Common method bias test

To assess potential common method variance (CMV), Harman’s single-factor test was conducted as an initial diagnostic procedure (Kock et al., 2021). The results indicated that the first unrotated factor accounted for 34.58% of the total variance, which is below the commonly recommended threshold of 40% (Harman, 1976). This preliminary finding suggests that no single factor dominated the covariance structure of the measurement items.

Additionally, variance inflation factor (VIF) values were examined in the structural model to detect potential collinearity effects, with all values remaining below conservative threshold levels (Hair et al., 2019). While these diagnostic approaches provide initial insights, common method bias cannot be fully ruled out through statistical tests alone. The cross-sectional design and reliance on self-report measures inherently involve some degree of method variance, which may influence the observed relationships. Further analyses using PLS-SEM diagnostics, including assessments of measurement model validity and construct discriminant validity, provided additional support for the robustness of the findings. The Fornell–Larcker criterion and heterotrait–monotrait (HTMT) ratios demonstrated adequate discriminant validity among constructs, thereby reducing concerns that fundamental measurement issues contributed to systematic bias.

### 4.2 Measurement model analysis

In this study, we conducted descriptive statistical analysis on the seven core variables involved demand, motivation, value, FoMO, PSE, PSR, and SVD, to assess the basic characteristics of the data and the reliability of the measurement model. The results are shown in Table 1. The mean value of each variable is between 3.35 and 4.09, and the standard deviation (Std. Dev.) is between 0.791 and 1.211, which indicate that the data distribution is relatively centralized, but some reasonable error exists.

Skew values are negative (−0.949 to −0.285) and Kurt values are between −0.847 and 1.329, which show that the data is left-skewed distribution, but most indicators generally conform to the assumption of normality (absolute skewness<3, absolute Kurt < 10). The factor loading of each measurement index is high (0.820 to 0.958) and the corresponding t value is significant (25.591 to 107.373), indicating that the index can effectively reflect its structure. In terms of internal consistency reliability, the Cronbach’s alpha coefficients (*α*) of all constructs were between 0.924 and 0.959, and the combined reliability (CR) were between 0.946 and 0.970. These results show that the measurement model of this study shows good characteristics in data distribution and reliability, and provides a reliable basis for subsequent analysis (Table 2).

**Table 2:** Descriptive statistics, reliability, and convergent validity (*N* = 825).

Variable	Indicator	Mean (std. dev.)	Skew.	Kurt.	Loading	*T*-values	*α*	CR
Need								
NE1		4.05 ± 0.868	−0.905	1.145	0.939	68.482	0.939	0.957
NE2		4.05 ± 0.860	−0.949	1.329	0.949	88.714		
NE3		3.95 ± 0.899	−0.714	0.473	0.882	71.934		
NE4		4.06 ± 0.829	−0.888	1.246	0.909	47.917		
Motive								
MO1		3.82 ± 0.899	−0.580	0.334	0.820	25.591	0.934	0.953
MO2		3.36 ± 1.211	−0.323	−0.847	0.938	57.814		
MO3		3.35 ± 1.207	−0.285	−0.832	0.947	78.280		
MO4		3.38 ± 1.173	−0.355	−0.749	0.945	60.065		
Value								
VA1		3.99 ± 0.823	−0.607	0.442	0.938	85.312	0.952	0.966
VA2		4.06 ± 0.799	−0.814	1.267	0.951	97.192		
VA3		3.97 ± 0.841	−0.561	0.302	0.924	91.255		
VA4		4.09 ± 0.791	−0.824	1.236	0.929	107.373		
Fear of missing out on short videos								
FoMO1		3.83 ± 0.902	−0.575	0.144	0.892	45.633	0.941	0.955
FoMO2		3.87 ± 0.858	−0.508	0.242	0.911	46.999		
FoMO3		3.80 ± 0.920	−0.465	−0.007	0.902	63.776		
FoMO4		3.96 ± 0.800	−0.568	0.566	0.909	56.707		
FoMO5		3.78 ± 0.945	−0.592	0.225	0.884	59.416		
Perceived Self-efficacy								
PSE1		3.56 ± 1.078	−0.511	−0.267	0.841	33.182	0.924	0.946
PSE2		3.84 ± 0.905	−0.686	0.611	0.931	37.883		
PSE3		3.87 ± 0.867	−0.498	0.105	0.920	37.334		
PSE4		3.72 ± 0.963	−0.551	0.075	0.917	46.564		
Perceived self-relevance								
PSR1		4.05 ± 0.829	−0.780	0.899	0.940	83.701	0.959	0.970
PSR2		4.06 ± 0.846	−0.909	1.218	0.952	103.834		
PSR3		4.02 ± 0.824	−0.747	0.890	0.958	97.230		
PSR4		3.98 ± 0.820	−0.600	0.457	0.925	69.597		
Short video dependence								
SVD1		4.00 ± 0.837	−0.653	0.500	0.912	54.691	0.928	0.949
SVD2		4.08 ± 0.796	−0.799	1.117	0.924	60.651		
SVD3		3.96 ± 0.824	−0.624	0.578	0.920	78.464		
SVD4		3.87 ± 0.853	−0.428	0.072	0.871	61.981		

NE, need; MO, motive; VA, value; FoMO, fear of missing out on short videos; PSE, perceived self-efficacy; PSR, perceived self-relevance; SVD, short video dependence.

As shown in Table 3, the average variance extracted (AVE) values of all constructs ranged from 0.809 to 0.891, exceeding the recommended threshold of 0.50, thereby indicating satisfactory convergent validity. Discriminant validity was assessed using the Fornell–Larcker criterion (Fornell and Larcker, 1981). The square roots of the AVE values (diagonal elements) were greater than the corresponding inter-construct correlations, suggesting adequate discriminant validity among the study variables. Together, these results indicate that the measurement model demonstrated acceptable convergent and discriminant validity, providing a sound basis for subsequent structural model analysis.

**Table 3:** Discriminant validity based on the Fornell–Larcker criterion.

Constructs	AVE	NE	MO	VA	FoMO	PSE	PSR	SVD
NE	0.846	**0.920**						
MO	0.835	0.443	**0.914**					
VA	0.875	0.863	0.502	**0.936**				
FoMO	0.809	0.714	0.699	0.782	**0.899**			
PSE	0.816	0.567	0.674	0.632	0.661	**0.903**		
PSR	0.891	0.892	0.473	0.711	0.748	0.594	**0.944**	
SVD	0.823	0.826	0.569	0.623	0.765	0.667	0.577	**0.907**

NE, need; MO, motive; VA, value; FoMO, fear of missing out on short videos; PSE, perceived self-efficacy; PSR, perceived self-relevance; SVD, short video dependence. Square roots of AVE values are shown in bold on diagonals.

To further verify the discriminant validity of the scale, we calculated the HTMT ratio of correlation. The HTMT values were further evaluated using a bootstrapping procedure with 5,000 samples to obtain 95% bias-corrected confidence intervals. As shown in Table 4, the HTMT values between all constructs ranged from 0.509 to 0.844, which were below the standard threshold of 0.90. Importantly, the bootstrap confidence intervals for the highest HTMT pairs (e.g., CPT and PSR: HTMT = 0.844, 95% CI [0.812, 0.879]) remained entirely below this threshold, providing robust statistical evidence for discriminant validity. While some constructs exhibited high inter-correlation, their HTMT confidence intervals did not include 1, and the constructs are theoretically distinct in their conceptual definitions and measurement items. Together with the Fornell–Larcker criterion, these results confirm that the latent variables in the measurement model possess satisfactory discriminant validity, laying a solid foundation for the subsequent hypothesis testing.

**Table 4:** Results of the HTMT test for assessing discriminant validity.

Constructs	NE	MO	VA	FoMO	PSE	PSR	SVD
NE							
MO	0.509						
VA	0.781	0.547					
FoMO	0.533	0.575	0.705				
PSE	0.659	0.603	0.584	0.732			
PSR	0.844	0.502	0.705	0.751	0.634		
SVD	0.708	0.614	0.735	0.649	0.728	0.690	

NE, need; MO, motive; VA, value; FoMO, fear of missing out on short videos; PSE, perceived self-efficacy; PSR, perceived self-relevance; SVD, short video dependence.

In this study, some constructs in the model exhibited relatively high correlations (e.g., the correlation coefficient between CPT and PSR was 0.892), which may reflect the close theoretical relationship between core personality traits and perceived self-relevance (Deci and Ryan, 2000). Despite the statistically high correlation, the constructs are clearly distinguishable in terms of theoretical definitions and measurement dimensions. For instance, CPT focuses on the integration of needs, motivations, and values, while PSR emphasizes the alignment between content and self-identity. Furthermore, the results of HTMT tests and Bootstrap confidence intervals support the discriminant validity of the constructs, thus eliminating the need to merge them or develop a higher-order model. The high explained variance (*R*^2^ = 0.894) primarily stems from the I-PACE model’s integration of multiple psychological mechanisms, rather than redundancy among constructs.

### 4.3 Descriptive statistics and correlations

Table 5 presents the descriptive statistics and intercorrelations among the primary study variables. The results indicated that all key constructs were significantly correlated in the expected directions (all *p* < 0.001). In general, CPT-related dimensions (need, motive, and value) showed moderate to strong positive correlations with FoMO, PSE, PSR, and SVD. These correlation patterns provide preliminary support for the hypothesized relationships and justify the subsequent structural equation modeling analyses.

**Table 5:** Intercorrelations for variables (*N* = 825).

	M ± SD	1	2	3	4	5	6	7
1. NE	4.03 ± 0.432	1						
2. MO	3.48 ± 0.565	0.403***	1					
3. VA	4.03 ± 0.407	0.861***	0.460***	1				
4. FoMO	3.85 ± 0.397	0.709***	0.673***	0.777***	1			
5. PSE	3.75 ± 0.478	0.553***	0.746***	0.619***	0.759***	1		
6. PSR	4.03 ± 0.415	0.889***	0.433***	0.910***	0.743***	0.583***	1	
7. SVD	3.98 ± 0.414	0.822***	0.534***	0.919***	0.863***	0.657***	0.873***	1

*p < 0.05, **p < 0.01, ***p < 0.001; NE, need; MO, motive; VA, value; FoMO, fear of missing out on short videos; PSE, perceived self-efficacy; PSR, perceived self-relevance; SVD, short video dependence.

### 4.4 Structural model analysis

Following prior research that adopted partial least squares structural equation modeling (PLS-SEM) to examine complex mediation mechanisms in psychological and educational contexts (Shi et al., 2025), the present study employed PLS-SEM to test the proposed serial mediation model. Prior to evaluating the structural relationships, multicollinearity among the predictor constructs was assessed. VIF values ranged from 1.21 to 2.74, which were well below the conservative threshold of 3.3 and the commonly used cutoff of 5, indicating that multicollinearity was not a concern.

The explanatory power of the structural model was evaluated using the coefficient of determination (*R*^2^). As shown in Table 6, CPT, FoMO, PSE, and PSR jointly explained 89.4% of the variance in SVD (*R*^2^ = 0.894). In addition, the *R*^2^ values for FoMO, PSR, and PSE were 0.403, 0.440, and 0.602, respectively, indicating substantial explanatory power for the endogenous constructs. Given the relatively high explained variance of SVD, the previously established low multicollinearity and satisfactory discriminant validity (HTMT < 0.85) suggest acceptable construct separation, thereby reducing concerns regarding redundancy-driven inflation.

**Table 6:** Results of calculating *R*^2^, *Q*^2^, and *f*^2^ effect sizes.

Endogenous construct			Predictor construct (*f*^2^)			
	*R* ^2^	*Q* ^2^	CPT	FoMO	PSE	PSR
FoMO	0.403	0.701	0.675			
PSE	0.602	0.541	0.375	0.350		
PSR	0.440	0.833	0.786			
SVD	0.894	0.841	0.405	0.422	0.113	0.381

NE, need; MO, motive; VA, value; FoMO, fear of missing out on short videos; PSE, perceived self-efficacy; PSR, perceived self-relevance; SVD, short video dependence.

To further assess the relative contribution of individual predictors, effect size (*f*^2^) values were examined. The results indicated that CPT (*f*^2^ = 0.405), FoMO (*f*^2^ = 0.422), PSR (*f*^2^ = 0.381), and PSE (*f*^2^ = 0.113) exerted medium to large effects on SVD, suggesting that the high explanatory power of the model was attributable to the substantive contributions of multiple predictors rather than statistical artifacts.

Predictive relevance was evaluated using the blindfolding procedure (Hair and Alamer, 2022). The *Q*^2^ values for all endogenous constructs were greater than zero (FoMO = 0.701, PSE = 0.541, PSR = 0.833, and SVD = 0.841), indicating adequate predictive relevance of the structural model.

**Figure 2:**
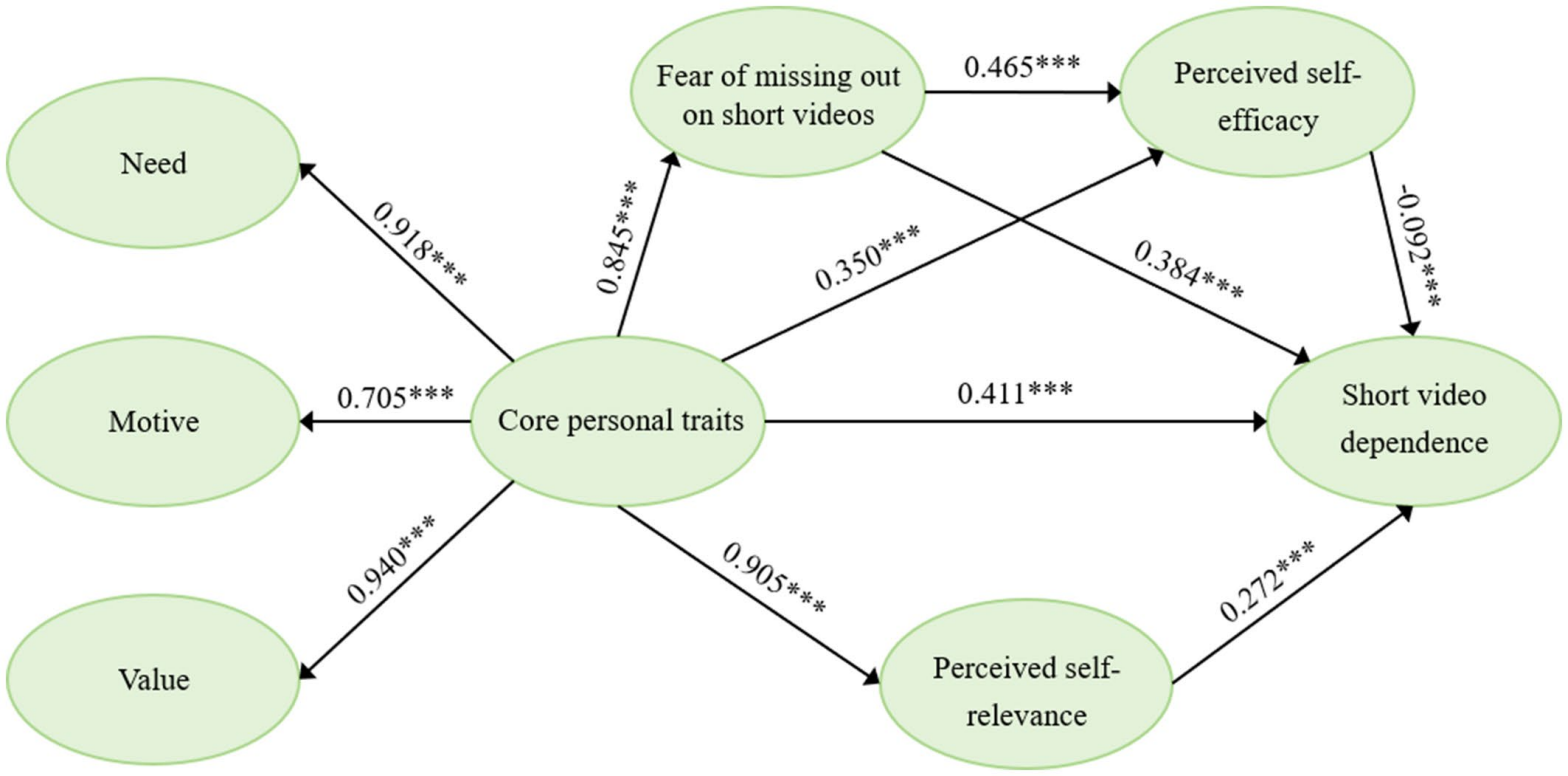
Structural model with standardized path coefficients.

To examine the significance of the hypothesized mediation effects, a nonparametric bootstrapping procedure with 5,000 resamples was conducted in SmartPLS. Standardized path coefficients and bias-corrected 95% confidence intervals were used to evaluate the significance of direct and indirect effects. Detailed results are reported in Table 6 and Figure 2.

Figure 2 illustrates the standardized path coefficients of the proposed structural model. The results indicated that CPT showed a significant positive association with SVD (*β* = 0.411, *p* < 0.001), such that individuals with stronger dispositional orientations toward short video use exhibited higher levels of problematic usage behaviors.

With respect to the indirect associations, CPT was significantly positively associated with FoMO, which was in turn positively associated with SVD, indicating that emotional concerns related to missing platform content statistically functioned as a partial mediator in the association between CPT and SVD. In addition, CPT showed a significant positive association with PSE. Importantly, we identified a statistically significant inconsistent mediation pattern (Zhao et al., 2010), the bivariate correlation between PSE and SVD was positive, while the specific indirect effect through PSE was negative (*β* = −0.092, *p* < 0.01). CPT was significantly positively associated with PSR, which was positively associated with SVD, highlighting the statistical contribution of self-related cognitive engagement to the overall link with platform use.

Furthermore, the statistical sequential pathway involving FoMO and PSE was statistically significant. Higher levels of FoMO were associated with increased perceived control over short video use, which was in turn linked to reduced levels of problematic engagement. This pattern may represent a form of perceived or illusory self-efficacy that co-occurs with heightened attention to platform-related information rather than reflecting actual differences in behavioral self-regulation.

The statistical model indicates that FoMO, PSE, and PSR statistically functioned as joint partial mediators in the association between CPT and SVD, underscoring the combined contributions of emotional arousal and cognitive self-evaluative processes to the link between CPT and SVD.

To examine the proposed serial mediation model, a bias-corrected percentile bootstrapping procedure with 5,000 resamples was conducted (see Table 7). The results indicated that the direct effect of CPT on SVD was statistically significant, with the 95% confidence interval not including zero (CI = [0.288, 0.534]), providing support for Hypothesis 1.

**Table 7:** Bootstrapping results for direct and indirect effects.

Path	Significance test of hypothesis				Bias-corrected 95%CI		Result
	Estimate	SE	*t*	*p*	LLCI	ULCL	
H1: CPT → SVD	0.411	0.065	5.321	***	0.288	0.534	Supported
H2: CPT → FoMO → SVD	0.324	0.041	4.154	***	0.246	0.403	Supported
H3: CPT → PSE → SVD	−0.032	0.012	3.012	**	−0.057	−0.031	Supported
H4: CPT → PSR → SVD	0.246	0.068	3.976	***	0.116	0.378	Supported
H5: CPT → FoMO → PSE → SVD	−0.036	0.011	3.201	**	−0.057	−0.015	Supported

*p < 0.05, **p < 0.01, ***p < 0.001. Bias-corrected 95% confidence intervals were obtained using 5,000 bootstrap resamples. NE, need; MO, motive; VA, value; FoMO, fear of missing out on short videos; PSE, perceived self-efficacy; PSR, perceived self-relevance; SVD, short video dependence.

Regarding the indirect effects, the mediation pathways through FoMO (CI = [0.246, 0.403]), PSE (CI = [−0.057, −0.015]), and PSR (CI = [0.116, 0.378]) were all statistically significant, supporting Hypotheses 2, 3, and 4, respectively. In addition, the sequential mediation pathway involving FoMO and PSE was also significant (CI = [−0.057, −0.015]), indicating that these two variables jointly mediated the relationship between CPT and SVD, thereby supporting Hypothesis 5.

Taken together, these findings suggest that the association between CPT and SVD is transmitted through multiple emotional and cognitive pathways, highlighting the complexity of the underlying psychological mechanisms.

It should be noted that the relatively high explanatory power of the model should be interpreted with caution. This result may partly reflect the conceptual proximity among psychological constructs, the context-specific nature of the measures, and shared self-report measurement characteristics. Nevertheless, additional model diagnostics, including discriminant validity assessment and predictive relevance indices, indicate that the observed explanatory power is not solely attributable to construct redundancy. Future research is encouraged to further examine model generalizability using independent samples, longitudinal designs, and alternative measurement approaches.

### 4.5 Robustness test

To verify the stability and reliability of the core findings and to rule out potential confounding effects of demographic and short-video use characteristics on parameter estimation, this study conducted a robustness test of the structural equation model by incorporating relevant covariates. Specifically, demographic variables (gender, age, grade, and major) and short-video use characteristics (frequency of use and duration per session) were included as control variables (Li and Zhang, 2023). A total of 5,000 bootstrap resamples were generated using AMOS to estimate robust standard errors and 95% confidence intervals. Standardized path coefficients were also reported. After controlling for the effects of these covariates on the core latent variables, the structural relationships among CPT, FoMO, PSE, PSR, and SVD were re-examined.

The results showed that, after controlling for covariates, the direct effect of CPT on SVD remained significant (*β* = 0.551, *p* < 0.001, 95% CI [0.409, 0.594]) and was consistent in direction with that observed in the baseline model (*β* = 0.411, *p* < 0.001, 95% CI [0.288, 0.534]). Moreover, the indirect effects of CPT on SVD via FoMO (*β* = 0.254, *p* < 0.001, 95% CI [0.134, 0.374]), via PSE (*β* = −0.048, *p* < 0.05, 95% CI [−0.113, −0.023]), and via PSR (*β* = 0.201, *p* < 0.001, 95% CI [0.198, 0.213]) all remained significant and directionally consistent with the baseline model. In addition, the serial mediation effect through CPT → FoMO → PSE → SVD was also significant (*β* = −0.031, *p* < 0.05, 95% CI [−0.055, −0.008]). With respect to covariate effects, only gender (*β* = −0.091, *p* < 0.01, 95% CI [−0.145, −0.037]) and frequency of short-video use (*β* = 0.042, *p* < 0.05, 95% CI [0.009, 0.076]) showed significant associations with FoMO. No other covariates exerted significant effects on the core latent variables. These results indicate that the core findings remain stable and reliable after controlling for covariates, demonstrating the robustness of the proposed model.

### 4.6 Analysis of inconsistent mediation patterns

Following the conceptual framework proposed by Zhao et al. (2010), additional analyses were conducted to examine the inconsistent mediation pattern identified in the proposed model. Inconsistent mediation occurs when indirect effects operating through different mediators have opposite signs, reflecting competing psychological mechanisms rather than statistical artifacts. In the serial mediation model, several competing pathways constituted a clear case of inconsistent mediation: an emotion-driven pathway (CPT → FoMO → SVD) with a positive indirect effect, a cognitively regulatory pathway (CPT → PSE → SVD) with a negative indirect effect, and a self-relevance pathway (CPT → PSR → SVD) with a positive indirect effect. As noted by Hayes (2018), inconsistent mediation patterns are theoretically meaningful because they indicate that a predictor can simultaneously function as both a trigger and a brake in the process leading to an outcome. The negative indirect effect through PSE, despite its positive bivariate correlation with SVD, suggests that perceived self-efficacy functions as a regulatory mechanism that counteracts emotion-driven tendencies toward short-video use.

## 5 Discussion and implications

While previous studies have primarily focused on the downstream socio-emotional consequences of SVD (Ye J. et al., 2025; Ye J.-H. et al., 2025), the present study extends this line of research by examining upstream personality-based vulnerability mechanisms and the cognitive–emotional pathways that lead to dependency.

### 5.1 The mediating role of fear of missing out on short video dependence

This study reveals associations consistent with a mediating role of FoMO in the link between CPT and SVD. Specifically, FoMO appears to attenuate the negative association between PSE and SVD while strengthening the positive association between CPT and SVD. Interestingly, the findings indicate that higher levels of FoMO are associated with higher levels of perceived self-efficacy, which contradicts previous research (Li et al., 2024; Servidio et al., 2024). This pattern may be related to the unique nature of the short-video context, in which students with high FoMO frequently use short-video platforms to rapidly access large volumes of information. They may misperceive their ability to rapidly scroll through videos as an indicator of self-efficacy, thereby developing an illusory sense of control (Akbari et al., 2024). Additionally, short-video platforms employ algorithmic reward mechanisms (e.g., likes, comments, and recommendations) to create a sense of control among users, thereby reinforcing this perceived efficacy (Wang et al., 2024). However, this interpretation requires further validation through experimental or longitudinal designs that directly assess illusory self-efficacy and distinguish it from actual self-regulatory capacity. Future research should incorporate behavioral measures of control (e.g., time-tracking tasks) alongside self-report scales specifically designed to capture overconfidence in media-use contexts. Furthermore, short-video recommendations rely on data-driven algorithmic systems that exacerbate information-bubble effects. These systems limit users to content aligned with their interests and foster a false sense of superiority (Sheng and Zhang, 2024). Notably, the FoMO-enhanced PSE observed in this study does not necessarily reflect genuine self-regulatory ability but may instead represent a byproduct of addictive engagement patterns. The statistical model indicates that the indirect effect via PSE (negative) and the indirect effect via FoMO (positive) operate in opposing directions in their associations with SVD.

The findings indicate that FoMO represents a significant associative pathway linking CPT to SVD. FoMO has been widely recognized as being significantly associated with problematic social media use, smartphone addiction, and SVD (Franchina et al., 2018; Hattingh et al., 2022). Dong et al. (2024) found that psychological stress and depression were positively associated with FoMO during the pandemic. This psychological mechanism has become a routine source of anxiety in adolescents’ digital lives. Adolescents fear missing out on rapidly changing online trends while simultaneously seeking to maintain virtual social identities through immediate engagement. CPT may be linked to heightened FoMO, which is in turn associated with anxiety and reduced life satisfaction, forming a cluster of risk factors related to higher SVD. For instance, individuals with higher levels of neuroticism are more prone to anxiety, restlessness, and negative emotions, making them more susceptible to FoMO. FoMO drives these individuals to use short-video platforms more frequently in an attempt to seek comfort and satisfaction, thereby temporarily alleviating negative emotions (Zhang et al., 2024).

### 5.2 The mediating role of perceived self-efficacy

This study indicates that CPT is positively associated with PSE, whereas PSE is negatively associated with SVD. This pattern is consistent with the view that PSE could functions as a protective factor linked to a reduced risk of SVD. While this pattern aligns with theoretical expectations regarding PSE as a protective factor, the positive association between CPT and PSE should be interpreted in light of the specific measurement approach adopted in this study. Previous studies have shown that individuals with high PSE are more likely to believe in their ability to control short-video use and resist excessive engagement (Zhao and Kou, 2024), suggesting that PSE is a key factor in understanding the association between CPT and SVD. According to Bandura (1992) SCT, individuals’ beliefs in their ability to successfully perform specific behaviors are key determinants of behavioral choices and effort investment. High PSE is generally associated with more adaptive coping strategies, whereas low PSE is linked to avoidance and less healthy behavioral patterns.

The CPT has been shown to be significantly associated with higher levels of PSE (Tsai et al., 2024). For instance, achievement motivation has been identified as a key predictor of general PSE (Kozłowska and Kuty-Pachecka, 2023). Individuals with high achievement motivation tend to set higher goals and invest greater effort in achieving them, thereby accumulating higher PSE through repeated success experiences. Furthermore, when individuals pursue goals that closely align with their core values, they are more likely to invest cognitive resources and emotional commitment, thereby strengthening their belief in goal attainment (Fan and Cui, 2024). In addition, existing research has shown that high PSE is positively correlated with psychological resilience and self-control (Wu et al., 2024). Greater psychological resilience and self-control reduce the influence of external stimuli, enabling individuals to maintain a positive mindset when facing pressure and challenges—an important factor in reducing SVD (Wang T. Y. et al., 2025; Wang X. et al., 2025). Moreover, PSE has been associated with greater learning engagement and reduced avoidance motivation, potentially helping students focus on tasks and reduce excessive short-video use (Ye et al., 2023). Taken together, these findings support the mediating role of PSE in the association between CPT and SVD.

The identification of inconsistent mediation patterns represents a key contribution of this study, as it reveals competing psychological processes operating in opposing directions rather than assuming unidirectional mechanisms. The findings support a dual-process conceptualization of short-video dependency, wherein automatic emotional responses (System 1) compete with deliberate cognitive regulation (System 2) (Krämer, 2014; Strack and Deutsch, 2004). The positive pathway via FoMO reflects an automatic, emotion-driven process that encourages impulsive platform engagement, whereas the negative pathway via PSE reflects a controlled regulatory process through which individuals consciously manage their usage behavior. As MacKinnon (2008) emphasized, inconsistent mediation patterns are particularly informative because they uncover complex underlying mechanisms that would otherwise be obscured in simple bivariate analyses. The simultaneous operation of these competing pathways helps explain why individuals often experience internal conflict in their media use, as they are simultaneously drawn to platforms by emotional forces while attempting to exert cognitive control.

### 5.3 The mediating role of perceived self-relevance

This study found that PSR is significantly associated with both CPT and SVD, suggesting that content perceived as self-relevant may contribute to dependency formation. This finding highlights the unique role of algorithmic personalization on short-video platforms. This finding is interpreted as evidence that platform-driven personalization may transform self-relevance from a positive psychological experience into a risk factor for excessive use. However, this interpretation requires further validation through experimental manipulation of self-relevance or examination of individual differences in resistance to algorithmic influence. Previous studies have primarily focused on emotional triggers (e.g., FoMO and pleasure) or behavioral reinforcement mechanisms. In contrast, the present study emphasizes the critical role of the “meaning-matching” cognitive process in the formation of media addiction, offering a novel psychological and cognitive perspective for understanding SVD. Specifically, CPT predisposes individuals to maintain heightened sensitivity and selectivity toward their external environment. As a result, individuals are more inclined to actively seek content that resonates with their life experiences, identity, or value systems. Short-video platforms leverage algorithmic recommendation systems to continuously capture user preferences and deliver highly personalized information streams, thereby reinforcing perceived alignment between content and self. This, in turn, strengthens users’ subjective perception of consistency between platform content and their personal identity (Przybylski et al., 2013). In this process, the platform transcends its role as a mere information provider and evolves into a mirrored space for self-identification and self-affirmation. PSR does not merely facilitate information matching; rather, it fosters a sense of recognition and reinforcement of self-meaning. Individuals who repeatedly encounter self-relevant content in short videos not only feel understood and seen but may also perceive the platform as a broader space for self-expression. This cognitive preference drives more frequent browsing, interaction, and sharing, while also unconsciously deepening emotional attachment and usage inertia toward the platform (Kelly et al., 2018).

Notably, this associative pathway differs from the one involving FoMO. It more accurately reflects the role of cognitive resonance and meaning construction in dependency formation. This aligns with recent perspectives on “digital media as an extension of the self,” illustrating that SVD involves not only excessive behavioral engagement but also processes through which individuals seek and affirm self-worth within the medium. However, media participation based on “meaning matching” is not neutral. SVD, as a highly malleable behavioral addiction pattern, reflects an implicit collusion between algorithmic systems and users’ cognitive structures. By repeatedly recommending “content related to you,” platforms create closed echo chambers that reinforce fixation on specific interests and identities, ultimately shifting media use from active exploration to passive immersion (Yang et al., 2023). Although this “self-centered” addictive pattern appears to satisfy psychological needs, it may ultimately weaken self-regulatory capacity and cognitive independence in real life. This finding resonates with recent research on media self-objectification and situational engagement theory, offering a psychological and cognitive explanation for individual differences in vulnerability to SVD.

### 5.4 Implications

This study provides a systematic analysis of the multidimensional psychological factors associated with SVD, clarifying the complex mediating pathways through which CPT influence FoMO, PSE, and PSR. The study incorporates the dynamic relationship between FoMO and PSE into the theoretical framework of SVD, validating their significant roles as sequential mediating mechanisms. It further expands the application of self-determination theory in the field of digital media dependence by emphasizing the synergistic influence of emotions and cognition on behavioral decision-making. Moreover, by focusing on the mediating effect of PSR, the study highlights the close connection between short-video platform content and users’ self-identity, providing a new theoretical perspective on the relationship between media use and identity construction. The mediating roles of FoMO and PSE further contribute to theoretical understanding of the relationship between CPT and SVD, indicating that the association between CPT and addictive behavior can be statistically explained by dual mediating pathways involving emotional activation and cognitive regulation. This study makes a significant methodological contribution by demonstrating how inconsistent mediation analysis can reveal complex psychological mechanisms in digital media research (Zhao et al., 2010). While traditional mediation analysis focuses on complementary mechanisms operating in the same direction, inconsistent mediation analysis enables the identification of competing mechanisms operating in opposite directions.

For college students, this study proposes a tiered intervention strategy. At the FoMO level, students should be guided to develop healthy social cognition and reduce reliance on virtual platforms through enhanced real-world social interactions (Yuan et al., 2021). At the PSE level, goal-management training and academic achievement feedback can enhance PSE and strengthen students’ ability to self-regulate short-video use (Bandura, 2004). At the PSR level, it is recommended to help learners break through information silos and expand cognitive boundaries through exposure to diverse content (Cinelli et al., 2021). These strategies not only provide concrete directions for intervening in college students’ media-use behavior but also offer a scientific basis for university mental health education and platform content optimization, thereby contributing to the development of a healthier digital learning environment.

## 6 Conclusion

Based on the I-PACE model, this study examines how Chinese college students’ CPT influence SVD through the serial mediation of FoMO, PSE, and PSR. The findings are consistent with a model in which CPT is both directly associated with SVD and indirectly linked to it via the sequential mediating paths of the aforementioned variables, thereby enriching the theoretical understanding of SVD mechanisms. However, this study has several limitations. First, the cross-sectional design limits the ability to infer causal relationships among variables, as it captures associations at a single point in time. Future research could adopt longitudinal designs with data collected at multiple time points to clarify the directional causal relationships among CPT, mediating variables, and SVD. Second, although this study employed Harman’s single-factor test as a preliminary assessment of common method bias, this diagnostic approach has recognized limitations. Future research could apply more rigorous methodological controls, such as marker variable techniques or latent method factor models, to more effectively isolate the influence of common method variance. Third, data collection relied primarily on self-report questionnaires, which may introduce response biases such as social desirability. Future research could integrate multiple data sources, including objective behavioral data (e.g., actual short-video usage duration tracked via applications) and qualitative interviews, to triangulate findings and enhance data credibility. Lastly, the sample was confined to college students in Henan Province, which may limit the generalizability of the findings to other populations or regions. Future studies should expand the sample to include students from diverse geographical regions and various types of institutions (e.g., vocational colleges and research-intensive universities) to enhance the external validity of the findings.

## Data Availability

The raw data supporting the conclusions of this article will be made available by the authors, without undue reservation.

## Footnotes

1
https://www.wjx.cn/
