# Supplementary material for: How do core personality traits influence short video dependence among Chinese college students? Evidence from a serial mediation analysis under the I-PACE model
Source: Front Psychol. 2026 Feb 25;17:1763608. doi: 10.3389/fpsyg.2026.1763608 (PMC12975990; doi:10.3389/fpsyg.2026.1763608)
Supplement: Supplementary file 1 [file Supplementary_file_1.docx]

Supplementary Material

Questionnaire Survey on Short Video Usage Behavior Among Chinese Youth 中国大学生短视频使用情况问卷调查

Dear user,

亲爱的用户：

Greetings! Thank you very much for taking the time to participate in this survey. Please read each question carefully and respond based on your actual situation and immediate impression. This questionnaire is intended to understand your usage behavior of mobile short video platforms.

您好！感谢您抽出宝贵时间参与本次问卷调查。请您认真阅读每一项问题，并根据您的实际情况和第一反应进行作答。本问卷旨在了解您在移动端使用短视频平台的行为特征。

Participation in this survey is entirely voluntary and anonymous. The data collected will be used solely for academic research purposes. The survey does not involve any personal privacy and will not negatively impact your health or well-being. If you feel any discomfort while answering, you may stop at any time, and your responses will not be recorded. Completion and submission of this questionnaire will be deemed as your informed consent.

本调查为完全自愿参与，采取匿名形式，所收集的数据仅用于学术研究，不涉及您的个人隐私，也不会对您的身心健康产生不良影响。如您在填写过程中感到不适，可以随时终止问卷作答，所填写内容将不被记录。若您完整填写并提交问卷，即视为您已知情同意参与本次调查。

Once again, we sincerely thank you for your cooperation and truthful responses.

再次感谢您认真的配合与如实的回答！

**（1） Basic Information 基本信息**

A. Your gender is：

您的性别是：

1. Male 男 2. Female 女

B. Your age (in years) falls within which of the following ranges：

您的年龄属于以下哪个范围：

1. ＜20 years old 小于20岁 3. 21-23 years old 21–23岁 4. ＞24 years old 大于24岁

C. What is your current academic year?

您的年级是：

1. Freshman 大一 2. Sophomore 大二 3. Junior 大三 4. Senior or above大四及以上

D. Your field of study is：

您所学的专业领域是：

1. Science 科学 2. Technology 技术 3. Engineering 工程 4. Mathematics 数学 5. Others

E. How often do you use mobile short-video apps (such as Douyin, wechat Video Account, etc.)?

您使用手机短视频应用（如抖音、微信视频号等）的频率是：

1. Occasionally 偶尔使用 2. Sometimes 有时使用 3. Often经常使用

F. The approximate time you spend using short videos each day (in hours) is:

您每天使用短视频的时间大约为（小时）：

1. ＜1h 小于1小时 2. 1-2h 1-2小时 3. 3-4h 3-4小时 4. ＞4h 超过4小时

**（2） Items specific information 项目具体信息**

| **Constructs** | **English version** | **Chinese version** | **Sources** |
| --- | --- | --- | --- |
| Core personal traits（CPT） |  |  | (Deci & Ryan, 2000; Przybylski et al., 2013) |
| Need (NE) | NE1. Using short video platforms gives me a sense of accomplishment when I discover useful content. | NE1.当我发现有用的内容时，使用短视频平台会给我一种成就感. |  |
|  | NE2. I feel more connected to others when I watch or comment on short videos. | NE2.当我观看或评论短视频时，我觉得与他人的联系更紧密. |  |
|  | NE3. I enjoy exploring content that matches my personal interests on short video platforms. | NE3.我喜欢在短视频平台上探索符合我个人兴趣的内容. |  |
|  | NE4. Watching short videos makes me feel like I’m part of a larger community. | NE4.看短视频，让我深切感受到自己属于一个更广阔的社会群体. |  |
| Motive (MO) | MO1. I watch short videos to relax and kill time when I’m bored. | MO1.当我无聊的时候，我看短视频来放松和消磨时间. |  |
|  | MO2. I watch short videos to follow what’s trending and stay up to date. | MO2.我刷短视频是为了跟随潮流，了解最新动态. |  |
|  | MO3. I use short videos as a way to express myself and share my interests. | MO3.我使用短视频来表达自己、分享我的兴趣. |  |
|  | MO4. I watch short videos to learn practical tips or knowledge. | MO4.我刷短视频是为了学习实用技巧或获取知识. |  |
| Value (VA) | VA1. Watching short videos helps me discover new ideas and think independently. | VA1.看短视频帮助我发现新的想法和独立思考. |  |
|  | VA2. I feel a sense of success when my short video content is liked or shared. | VA2.当我的短视频内容被点赞或分享时，我有一种成功的感觉. |  |
|  | VA3. I value short videos that allow me to help or inspire others. | VA3.我很看重能让我帮助或激励他人的短视频. |  |
|  | VA4. I watch short videos because I enjoy excitement and new experiences. | VA4.我看短视频是因为我喜欢刺激和新的体验. |  |
| Fear of missing out on short videos  (FoMO) |  |  | (Przybylski et al., 2013) |
|  | FoMO1. I feel anxious when I haven’t checked short video platforms for a while | FoMO1.一段时间不刷短视频我会感到焦虑. |  |
|  | FoMO2. I feel left out when I miss trending short video content. | FoMO2.错过热门短视频内容时我感到被排除在外. |  |
|  | FoMO3. I often feel the need to catch up on the latest short video trends. | FoMO3.我常常觉得需要赶上最新的短视频潮流. |  |
|  | FoMO4. I get uneasy when I don’t know what’s trending on short video platforms. | FoMO4.我不知道短视频平台上流行什么时会感到不安. |  |
| Self-Efficacy (SE) |  |  | (García-Salirrosas et al., 2025; Pumptow & Brahm, 2021) |
|  | SE1. I believe I can control the amount of time I spend watching short videos each day. | SE1.我相信自己可以控制每天观看短视频的时间. |  |
|  | SE2. Even when highly engaging content is recommended, I am able to stop using short-video apps promptly. | SE2.即使短视频平台推送我感兴趣的内容，我也能及时关闭应用程序. |  |
|  | SE3. I am confident that I can avoid using short video apps when I need to study or work. | SE3.我有信心在学习或工作需要时不打开短视频应用. |  |
|  | SE4. I trust my ability to set—and stick to—a reasonable daily limit for short-video use. | SE4.我相信自己可以设定并坚持每天使用短视频的合理时间上限. |  |
|  | SE5. I can refrain from relying on short videos to cope with boredom or negative emotions. | SE5.在情绪低落或无聊的时候，我也能避免依赖短视频来打发时间. |  |
| Perceived self-relevance (PSR) |  |  | (Klimmt et al., 2018; Lee & Watkins, 2016) |
|  | PSR1. The short videos I watch typically reflect my personal interests. | PSR1.我观看的短视频通常反映了我的个人兴趣. |  |
|  | PSR2. I see parts of myself in the people or scenarios shown in short videos. | PSR2.我能在短视频中的人物或情境中看到自己的影子. |  |
|  | PSR3. Short video content often aligns with my personal values or beliefs. | PSR3.短视频内容常常与我的价值观或信念一致. |  |
|  | PSR4. I feel emotionally connected to many of the short videos I watch. | PSR4.我与很多短视频内容有情感上的联系. |  |
| Short video dependence (SVD) |  |  | (Ye et al., 2025) |
|  | SVD1. I find it hard to stop watching short videos, even when I need to do something else. | SVD1.即使需要做其他事情，我也很难停止刷短视频. |  |
|  | SVD2. I often spend more time watching short videos than I originally planned. | SVD2.我经常花比计划更多的时间刷短视频. |  |
|  | SVD3. After watching short videos, I find it difficult to concentrate on studying or other tasks. | SVD3.看完短视频后，我很难集中精力学习或处理其他任务. |  |
|  | SVD4. I have tried to reduce my short video usage but failed. | SVD4.我尝试过减少刷短视频的时间，但没有成功. |  |

**Reference**

Deci, E. L., & Ryan, R. M. (2000). The "what" and "why" of goal pursuits: Human needs and the self-determination of behavior. *Psychological Inquiry*, *11*(4), 227-268. <https://doi.org/10.1207/S15327965PLI1104_01>

García-Salirrosas, E. E., Millones-Liza, D. Y., Rondon-Eusebio, R. F., Esponda-Pérez, J. A., Salas-Tenesaca, E. E., Armas-Herrera, R., & Zumba-Zúñiga, M. F. (2025). The Interaction Between Self-Efficacy, Fear of Failure, and Entrepreneurial Passion: Evidence from Business Students in Emerging Economies. *Behavioral Sciences*, *15*(7), 951. <https://doi.org/10.3390/bs15070951>

Klimmt, C., Hefner, D., Reinecke, L., Rieger, D., & Vorderer, P. (2018). The permanently online and permanently connected mind: Mapping the cognitive structures behind mobile internet use. In.

Lee, J. E., & Watkins, B. (2016). YouTube vloggers' influence on consumer luxury brand perceptions and intentions. *Journal of Business Research*, *69*(12), 5753-5760. <https://doi.org/10.1016/j.jbusres.2016.04.171>

Przybylski, A. K., Murayama, K., DeHaan, C. R., & Gladwell, V. (2013). Motivational, emotional, and behavioral correlates of fear of missing out. *Computers in Human Behavior*, *29*(4), 1841-1848. <https://doi.org/10.1016/j.chb.2013.02.014>

Pumptow, M., & Brahm, T. (2021). Students’ Digital Media Self-Efficacy and Its Importance for Higher Education Institutions: Development and Validation of a Survey Instrument. *Technology, Knowledge and Learning*, *26*(3), 555-575. <https://doi.org/10.1007/s10758-020-09463-5>

Ye, J., Wang, W., Huang, D., Ma, S., Chen, S., Dong, W., & Zhao, X. (2025). Short video addiction scale for middle school students: development and initial validation. *Scientific Reports*, *15*(1), 9903. <https://doi.org/10.1038/s41598-025-92138-x>
